# Interplay of Dyadic Consensus, Reflective Functioning, and Perinatal Affective Difficulties in Modulating Fear of COVID-19 among First-Time Mothers: A Mediation Analysis

**DOI:** 10.3390/ijerph21070848

**Published:** 2024-06-28

**Authors:** Andrea Fontana, Sonia Mangialavori, Grazia Terrone, Lucrezia Trani, Eleonora Topino, Valeria Trincia, Giulia Lisi, Giuseppe Ducci, Marco Cacioppo

**Affiliations:** 1 Department of Human Sciences, LUMSA University, 00193 Rome, Italy; l.trani@lumsa.it (L.T.); e.topino@lumsa.it (E.T.); m.cacioppo@lumsa.it (M.C.); 2 Department of Pathophysiology and Transplantation, University of Milano, 20122 Milan, Italy; sonia.mangialavori@unimi.it; 3 Department of History, Cultural Heritage, Education and Society, University of Rome Tor Vergata, 00133 Rome, Italy; grazia.terrone@uniroma2.it; 4 Department of Mental Health, ASL Roma 1, 00193 Rome, Italy; valeria.trincia@aslroma1.it (V.T.); giulia.lisi@aslroma1.it (G.L.); giuseppe.ducci@aslroma1.it (G.D.)

**Keywords:** fear of COVID-19, dyadic adjustment, perinatal affective symptoms, reflective functioning, pregnant women

## Abstract

Background: The COVID-19 pandemic has exacerbated fears and anxieties, potentially influencing maternal perinatal mental health. This study addresses a gap in the literature on fear of COVID-19 in pregnant women, aiming to identify contributing factors. Method: Participants were 401 primiparous women with an average age of 34 years (SD = 4.56) who were recruited through the National Health System during birth support courses. They completed a series of self-reported instruments via an online survey, providing information on their levels of reflective functioning, dyadic consensus, perinatal maternal affectivity, and fear of COVID-19. Pearson’s correlation and mediation analysis via a generalized linear model were implemented to analyze the collected data. Results: The relationship between dyadic consensus and fear of COVID-19 was significant and negative. Furthermore, a significant parallel mediation involving perinatal maternal affectivity and reflective functioning was found to the extent that, when these factors were inserted into the model, the direct association between dyadic consensus and fear of COVID-19 became non-significant (total mediation). Conclusions: The results highlight the importance of dyadic adjustment in alleviating maternal COVID-19 fear, emphasizing interventions promoting couple functioning, mentalization, and addressing affective difficulties. Such approaches are vital for supporting expecting mothers during challenging times like the COVID-19 pandemic.

## 1. Introduction

The transition to parenthood is widely recognized as a sensitive evolutionary stage, closely associated with an increase in psychological vulnerability [1]. Moreover, the challenges posed by the COVID-19 pandemic have added an extra layer of complexity to this transition, exacerbating feelings of uncertainty and fear among expectant and new parents [2]. Early studies initially suggested that COVID-19 posed no significantly greater risk to pregnant women, or their fetuses compared to non-pregnant individuals [3]. However, recent research indicates that while infection rates in pregnant women mirror those of the general population, the consequences can be more severe, including higher rates of severe illness, hospitalization, and ICU admissions [4,5]. Although instances of in utero infection and vertical transmission of COVID-19 are rare, contracting the virus during pregnancy heightens the risk of complications such as preeclampsia, preterm birth, and low birth weight [6,7]. These findings underscore the significant health risks posed by COVID-19 to pregnant women and their babies, highlighting the fear experienced by expectant mothers during the pandemic.

Schimmenti, Billieux, and Starcevic [8] introduced a theoretical framework to explain fear experiences during the COVID-19 pandemic. They suggested that a pandemic could trigger fears encompassing key psychological mechanisms for understanding reality. Consequently, fear experiences during a pandemic involve bodily, relational, cognitive, and behavioral dimensions, which are interconnected. Moreover, the framework proposes that these dimensions of fear are not arranged hierarchically; instead, they are structured in a dialectical manner: the bodily dimension encompasses fear related to the body/fear for the body; the relational dimension involves fear related to others/fear for others; the cognitive dimension includes fear related to knowing/fear of not knowing; and the behavioral dimension encompasses fear related to taking action/fear of inaction.

In addition to the fear of contracting COVID-19, pregnancy introduces specific anxieties for women. Fear of COVID-19 may exacerbate certain pregnancy-specific fears, such as worry about the fetus’s health or the mother’s own health and worry about going to the hospital [9,10]. While studies indicate that fear and anxiety stemming from illness can enhance individuals’ adherence to preventive behaviors, heightened fear of COVID-19 has been directly linked to mental health issues [11]. In their study, Ahorsu et al. revealed a significantly negative correlation between pregnant women’s fear of COVID-19 and their mental quality of life; the greater the fear, the lower the mental quality of life.

Pregnant women’s fears and concerns encompass various factors, including sociomedical and socioeconomic aspects, fetal and maternal health, and relational issues [12]. Among relationship factors, some studies have found that perceived a good marital adjustment was inversely related to childbirth-related fear [13], maternal psychological distress during the pandemic [14], and the risk of developing prenatal affective symptoms [15,16]. Specifically, in their study, Mangialavori et al. [16] found that among the dimensions of marital adjustment, dyadic consensus—which refers to the agreement between partners regarding marital decisions or daily life, as well as their experiences of affection in the relationship—appears to be the most significant relational factor, along with affective expression and support from the partner, in predicting prenatal maternal depression.

Reflective functioning, or the ability to understand and reflect on one’s own and others’ mental states, is crucial for effective interpersonal relationships and self-regulation under stress. In the context of pregnancy, particularly during the unprecedented challenges posed by the COVID-19 pandemic, this capacity becomes even more significant. Reflective functioning allows expectant mothers, especially first-timers who are navigating the anxieties of impending motherhood without prior experience, to better manage their fears and uncertainties [17]. Studies have shown that reflective functioning in individuals can mitigate the psychological impact of stress, enhancing resilience and coping mechanisms [18]. For first-time expectant mothers, who may face heightened vulnerability to perinatal affective disturbances, the role of reflective functioning is particularly pivotal. It not only aids in managing personal fears related to health and well-being but also facilitates better communication with partners, thereby strengthening dyadic relationships and overall support systems during the pandemic [19]. Some authors have also found that support from both partners and family is the strongest predictor of maternal reflective functioning, which entails the ability to imagine thoughts, feelings, needs, and intentions within oneself and others [20,21]. A high-quality supportive partner relationship appears to facilitate adaptation to impending motherhood [22], resulting in stable perceptions of the future infant in the last trimester of pregnancy [23]. Feeling supported makes it easier for women to gain confidence in their new self-perceptions and eases the transition to motherhood [24], especially during times of fear and high uncertainty such as the COVID-19 pandemic.

Given these points, while numerous studies have examined the impact of the fear of COVID-19 on the mental health of pregnant women [10,11,25], there is currently a gap in the literature regarding the specific factors contributing to this fear. In this regard, the main aim of this study was to investigate the factors contributing to the fear of COVID-19 among pregnant women, considering the role played by perceived dyadic consensus and potential mediators such as maternal reflective functioning and perinatal affective difficulties. In line with Schimmenti et al.’s conceptual framework [8], our study examines the intricate interplay between bodily, relational, cognitive, and behavioral domains of fear among first-time expectant mothers. Reflective functioning, which is a part of the cognitive domain, could mitigate the intensity of fear by providing a better understanding and management of pandemic-related stressors. Additionally, perceived couple consensus by mothers is examined as part of the relational domain, focusing on how perceived support by mothers within the couple can influence emotional well-being during pregnancy. Furthermore, this study delves into the direct impact of the COVID-19 pandemic on expectant mothers by measuring the fear associated with the virus, which falls into the bodily domain of Schimmenti et al.’s model [8]. Lastly, perinatal affective disorders are explored to understand their intersection across these domains, particularly affecting cognitive, bodily, and interpersonal aspects by influencing both mental health and perceptions of bodily integrity during pregnancy. By integrating these variables within the established framework by Schimmenti et al. [8], our study aims to provide a comprehensive understanding of the dynamics at play in shaping the pandemic experience for expectant mothers. This approach not only adheres to but enriches the theoretical model by applying it to a specific, highly impacted population, thus offering significant insights into the multifaceted nature of fear and its management during the unprecedented times of the COVID-19 pandemic. This exploration is crucial for developing targeted interventions that can support maternal mental health more effectively during and beyond the pandemic crisis.

## 2. Method

### 2.1. Participants and Procedure

The study sample comprised 401 primiparous women with a mean age of 34 years (SD = 4.56, range: 22–48 years). Participants, in the third trimester of pregnancy, were recruited through the ASL Rome 1 Mental Health District, National Health System (NHS), in the metropolitan city of Rome (Italy) at the beginning of the birth support courses, which were offered in a video-synchronous format due to the COVID-19 pandemic restrictions. Eligibility criteria included being pregnant with one’s first child, proficiency in Italian, being in the third trimester of pregnancy, and consent to participate. All procedures, including informed consent, were carried out online, ensuring participant privacy. Data were collected via an online platform in 2022, during the latter stages of the COVID-19 pandemic, following the full recommendation and implementation of the vaccination campaign for pregnant women. This period was marked by an evolving scientific consensus on the safety and efficacy of COVID-19 vaccinations for pregnant women, which influenced their perceptions and decision-making processes regarding vaccination during any trimester of pregnancy. Upon accessing the platform through an anonymous link, participants were required to review and endorse an informed consent form before proceeding with the survey. Completing the survey typically required approximately 20 min. Throughout the recruitment and survey administration phases, we emphasized the voluntary nature of participation and ensured the confidentiality and anonymity of all respondents. To ensure a complete dataset, respondents were required to answer all questions, thereby precluding any instances of missing data. The present study is an extension of a study on risk factors for perinatal maternal affective disorders approved by the Ethics Committee CEIIAV (Ethical Committee IRST IRCCS AVR, Mendola, Italy, 1607/16516). Thus, this study was conducted in accordance with the ethical principles contained in the Helsinki Declaration and following the ethical requirements established by the National Board of Italian Psychologists Code of Ethics for the Psychologist.

### 2.2. Measures

Fear of COVID-19: Fear of COVID-19 was quantitatively evaluated using the Multidimensional Assessment of COVID-19-Related Fears (MAC-RF [26]). The MAC-RF, a self-reported instrument, is designed to measure various domains of fear associated with the COVID-19 pandemic. Participants rated items on a five-point Likert scale, from 0 (“very unlike me”) to 4 (“very like me”), to reflect their degree of fear. The scale includes prompts like “I am frightened about my body being in contact with objects contaminated by the coronavirus” addressing fears related to one’s physical well-being, and “I fear that people who are around me can infect me” addressing the fear of interpersonal transmission. The MAC-RF encompasses both physical and social facets of COVID-19-related fears. Elevated scores on the MAC-RF signify increased fear levels, providing insights into the potential impacts on individuals’ mental health and well-being during the pandemic [23]. In the present study, the MAC-RF’s reliability was deemed satisfactory, evidenced by McDonald’s ω and Cronbach’s α both at 0.83.

Couple Functioning: Couple adjustment was measured using the Dyadic Adjustment Scale (DAS; [27,28]), a 32-item instrument assessing the quality of couple relationships across different dimensions. The DAS is composed of four subscales, each addressing a specific aspect of couple dynamics: dyadic consensus (extent of agreement on relationship matters), dyadic satisfaction (level of contentment within the relationship), dyadic cohesion (degree of closeness and shared activities), and affective expression (frequency of expressions of affection and sexual interaction). For the purposes of this study, the focus was placed exclusively on dyadic consensus. Scores on this subscale are directly proportional to the degree of agreement between partners, with higher scores signifying greater harmony and the future mother’s greater perceived support. The Italian adaptation of the DAS demonstrated robust internal consistency within our sample, with a McDonald’s ω of 0.86 and a Cronbach’s α of 0.84 for the dyadic consensus subscale.

Perinatal Maternal Affectivity: Perinatal maternal affectivity was assessed using the Perinatal Assessment of Maternal Affectivity (PAMA; [29]). Conceived as the counterpart to the Perinatal Assessment of Paternal Affectivity (PAPA; [30]), the PAMA is a self-reported instrument designed to evaluate a broad range of affective experiences during the perinatal period. It comprises seven items, each rated on a four-point Likert-type scale from 0 (“Not at all”) to 3 (“A lot”), to quantify the severity of various symptoms and behaviors reported in the preceding two weeks. The PAMA’s unidimensional model encapsulates diverse psychological dimensions, including anxiety, depression, perceived stress, irritability/anger, relationship difficulties, somatization, and physiological issues. Higher scores on the PAMA denote increased perinatal maternal affective difficulties. The PAMA demonstrated robust positive correlations with indicators of depressive symptoms, psychological distress, and perceived stress, alongside a negative correlation with dyadic adjustment scores [26]. The internal consistency of the PAMA in this study was confirmed as satisfactory, with a McDonald’s ω of 0.79 and a Cronbach’s α of 0.80.

Reflective Functioning: The Reflective Functioning Questionnaire (RFQ-8; [31,32]) employs a seven-point Likert scale across eight items to gauge an individual’s mentalizing capacity, where higher scores denote greater difficulty in accurately interpreting one’s own and others’ mental states. This assessment is particularly focused on the individual’s insight into their own behaviors. Research has indicated a strong correlation between the RFQ and personality pathology, and its association with constructs such as emotional lability and impulsivity has been noted. For instance, one item states “Sometimes I do things without really knowing why” capturing the essence of mentalizing challenges. In the present study, the RFQ-8 demonstrated robust psychometric properties, with a McDonald’s omega of 0.80 and a Cronbach’s alpha of 0.79, confirming its internal consistency and reliability in the evaluation of reflective functioning.

### 2.3. Analytic Strategy

Data analyses were performed using SPSS v.24 and Jamovi v.2.3.19. The initial phase involved a thorough examination of the item pool for accuracy in data entry and the identification of missing values. Multivariate outliers were identified using Mahalanobis distance and assessed for significance with a χ^2^ test, applying a threshold of *p* < 0.001. The identified outliers were excluded from subsequent analyses [33]. Descriptive statistics for all study variables were subsequently calculated (Table 1). Then, the normality of distribution for each variable was appraised by scrutinizing skewness and kurtosis indices. Subsequently, Pearson’s correlation coefficients were then computed to determine the associative directions among the variables under study.

**Figure 1:**
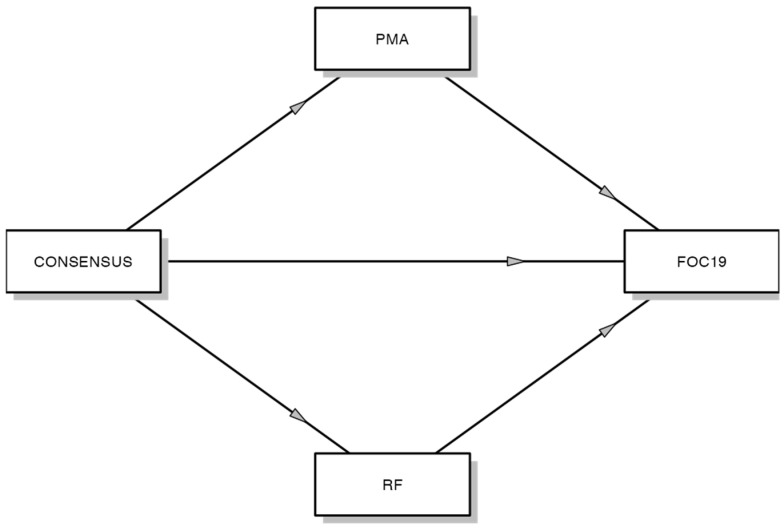
The mediation of perinatal maternal affectivity and reflective functioning in the relationship between dyadic consensus and fear of COVID-19. Note: RF = reflective functioning (Reflective Functioning Questionnaire; RFQ-8); CONSENSUS = dyadic consensus (Dyadic Adjustment Scale; DAS); PMA = perinatal maternal affectivity (Perinatal Assessment of Maternal Affectivity; PAMA); FOC19 = fear of COVID-19 (Multidimensional Assessment of COVID-19-Related Fears; MAC-RF).

The core of the analytic strategy entailed conducting a mediation analysis via a generalized linear model (GLM). This analysis probed the direct and indirect effects of dyadic consensus on maternal fear of COVID-19, considering perinatal affective maternal difficulties and reflective functioning as potential mediators (Figure 1). Two mediation pathways were investigated: (a) the influence of dyadic consensus on maternal fear of COVID-19 through perinatal affective maternal difficulties and (b) the impact of dyadic consensus on maternal fear of COVID-19 through reflective functioning difficulties. The comprehensive model evaluated the combined effects of perinatal affective maternal difficulties, reflective functioning, and dyadic consensus on maternal fear of COVID-19. For the mediation analysis, Fritz and MacKinnon’s simulation study was employed to determine the minimum sample size required to detect the mediated effect [34]. As per Fritz and MacKinnon, a sample size of n = 391 is necessary to achieve 80% power. Thus, the obtained sample size of n = 401 is adequate for examining the study’s hypotheses.

## 3. Results

A significant majority of the women had completed higher education, with 75.9% holding a university degree. High school diplomas were reported by 23.8% of the sample, and 0.3% had middle school education as their highest attainment. In terms of marital status, 48.7% of participants were cohabitating, 44.8% were married, 0.5% were separated, and 6.0% were single. Regarding mental health history, 31.7% of the participants reported experiencing significant stressors or mental health issues, such as depression or anxiety, in the recent past, while 68.3% reported no such experiences.

Missing data analysis confirmed the dataset was complete, with no instances of missing data. However, multivariate outliers (n = 19) were detected and, to preserve the integrity of the analysis as per Tabachnick and Fidell [33], these cases were excluded. Thus, the final sample comprises 382 women. Pearson’s correlation analysis revealed significant relationships between study variables. Specifically, Table 2 illustrates that fear of COVID-19 exhibited a positive correlation with both perinatal maternal affective difficulties and challenges in reflective functioning. Conversely, a negative correlation was observed between dyadic consensus and fear of COVID-19, suggesting that higher levels of perceived dyadic functioning in future mothers are associated with reduced fear of COVID-19. Additionally, dyadic consensus was found to be negatively associated with perinatal affective maternal difficulties. This indicates that future mothers perceiving greater consensus within their relationships tend to report fewer perinatal maternal affective difficulties.

The analysis revealed significant indirect effects for both pathways (see Table 3). The path from dyadic consensus through perinatal affective maternal difficulties to fear of COVID-19 was significant, indicating that higher couple consensus is associated with reduced perinatal affective maternal difficulties, which in turn leads to lower maternal fear of COVID-19 (*β* = −0.057, *SE* = 0.0256, 95% CI [−0.1040, −0.0035], *p* = 0.036). Similarly, the indirect effect from dyadic consensus through reflective functioning to fear of COVID-19 was significant, suggesting that improved couple consensus is linked to better reflective functioning, thereby reducing maternal fear of COVID-19 (*β* = −0.046, *SE* = 0.0187, 95% CI [−0.0807, −0.0076], *p* = 0.018). Interestingly, the direct effect of dyadic consensus on fear of COVID-19 was not significant (*β* = −0.0159, *SE* = 0.0567, 95% CI [−0.1262, 0.0959], *p* = 0.789), indicating that the relationship between maternal perceived dyadic consensus and maternal fear of COVID-19 is completely mediated through the effects on perinatal affective maternal difficulties and reflective functioning. The total effect of dyadic consensus on fear of COVID-19 was significant (*β* = −0.118, *SE* = 0.0486, 95% CI [−0.2082, −0.0179], *p* = 0.020), underscoring the overall importance of couple adjustment in mitigating maternal fear of COVID-19.

## 4. Discussion

In light of the extensive research concerning the impact of the fear of COVID-19 on the mental health of pregnant women, a notable gap exists in understanding the specific determinants underlying this fear. Therefore, the primary aim of this study was to explore the factors contributing to the fear of COVID-19 among pregnant women. In pursuit of this objective, our investigation focused on elucidating the role of dyadic consensus perceived by mothers while also examining potential mediators such as maternal reflective functioning and perinatal affective difficulties.

Our findings underscore the importance of dyadic consensus perceived by mothers as a significant predictor in understanding the fear of COVID-19 among pregnant women. Indeed, research suggests that the fear of COVID-19 among pregnant women is influenced not only by individual factors such as personal concerns regarding their health and that of their baby and information-seeking behaviors but also by relational dynamics within their romantic couple [35,36,37]. In the context of pregnancy, the support and understanding of a partner play a crucial role in shaping a woman’s experience and perception of the pandemic [35]. Dyadic consensus can manifest in multiple ways. Firstly, it involves shared perceptions of the threat posed by COVID-19 and agreement on appropriate preventive measures. When partners are aligned in their understanding of the risks and precautions, pregnant women may experience less anxiety and feel more supported in managing their concerns [35]. Secondly, dyadic consensus encompasses shared coping strategies and support mechanisms. Partners who agree about how to navigate challenges related to COVID-19, such as adapting to changes in prenatal care or addressing concerns about childbirth, can provide valuable emotional and practical support to each other, thereby mitigating the fear and stress experienced by pregnant women [14,38]. Moreover, the quality of communication and collaboration within the partnership influences dyadic consensus [27]. Open and empathetic communication allows couples to express their fears, seek reassurance, and make joint decisions about protective measures and healthcare choices. Conversely, a lack of consensus or discord within the couple may exacerbate the fear of COVID-19 among pregnant women, leading to heightened anxiety and uncertainty.

Furthermore, our analysis suggests that maternal reflective functioning and perinatal difficulties may mediate the link between dyadic consensus and fear of COVID-19, revealing the intricate pathways through which dyadic consensus impacts this fear. Specifically, perinatal affective difficulties encompass emotional and psychological challenges experienced by mothers during pregnancy and the postpartum period, such as depression or anxiety. These challenges may substantially affect a mother’s ability to navigate the uncertainties of the pandemic [25]. The perception of a strong dyadic consensus alleviates these perinatal maternal difficulties [15], enhancing the mother’s capacity to cope with pandemic-related stressors and ultimately reducing her fear of COVID-19. Moreover, our results showed that high levels of couples’ perceived consensus by mothers correlated with better reflective functioning in expectant mothers, which in turn reduced their fear of COVID-19. This implies that when expectant mothers feel supported and understood by their partners, it positively influences their ability to reflect on and understand their own thoughts, emotions, and behaviors, as well as those of their partners [39,40]. This enhanced reflective functioning likely fosters a greater sense of emotional stability and resilience in pregnant women when facing stressors [21] such as the fear of COVID-19, thereby diminishing the likelihood of experiencing overwhelming fear or anxiety related to the pandemic.

Overall, our study underscores the complex interplay between interpersonal dynamics, individual psychological factors, and the fear of COVID-19 among pregnant women. By addressing these multifaceted influences, clinicians and public health professionals can develop comprehensive and tailored interventions to support the mental health and well-being of expectant mothers during these challenging times. Our findings highlight how the interplay between these domains affects first-time expectant mothers during the COVID-19 pandemic, providing empirical support for the comprehensive framework proposed by Schimmenti et al. [8]. This theoretical alignment has allowed for a nuanced understanding of the multifaceted nature of fear and its management during such unprecedented times. 

The results suggest that interventions focused on improving couple dynamics, particularly increasing their consensus on matters important to their relationship, could effectively reduce maternal fear of COVID-19. These interventions could target perinatal affective maternal difficulties and enhance reflective functioning by promoting open communication, mutual understanding, and shared decision-making between partners [39]. Indeed, in their recent study, Berthelot et al. [19] discovered that a brief intervention targeting the psychological well-being of pregnant women led to improved couple functioning, reflected in increased satisfaction levels, improved communication, and heightened interest in the partner’s psychological state. This finding provides significant evidence for the positive impact of mentalization-based interventions on important attachment relationships. By enhancing prenatal couple dynamics, the intervention may pave the way for effective coparenting and emotional stability at home, fostering a positive, supportive, and reflective environment for infant development.

Although this is the first study to examine the associations between perceived dyadic consensus and fear of COVID-19 during pregnancy and to explore maternal perinatal difficulties and reflective functioning as the potential mechanisms linking couple dynamics and fear related to the pandemic in pregnant women, there are some limitations that should be acknowledged. The main limitation of the present study was its cross-sectional design, which precludes conclusions about causality. Indeed, all findings are correlational in nature and cannot be considered predictive (see Hayes [41] for counterargument regarding mediation models). Follow-up studies are necessary to monitor changes in fear of COVID-19, dyadic adjustment, maternal perinatal difficulties, and reflective functioning over the course of the pandemic and in the aftermath. Secondly, although our exploration of these specific mediating factors offers new perspectives, the overall topic of fear related to COVID-19 has been extensively studied. This might limit the perceived novelty of our findings to those already familiar with the extensive literature on pandemic-related anxieties. Convenience sampling is another limitation of this study that reduces the generalizability of the results. Furthermore, data were exclusively collected through self-report questionnaires, validated within an Italian context. However, relying solely on self-report data may not adequately capture the complexity of these processes. Future research should integrate diverse data collection methods, including clinical interviews or diaries, to enhance understanding. Implementing a mixed methods approach could strengthen the reliability of findings and offer a more comprehensive insight into the intricate interplay between dyadic consensus, maternal perinatal difficulties, reflective functioning, and fear of COVID-19 among first-time expectant mothers [42]. Additionally, this study focused exclusively on expectant mothers, which inherently limits our exploration of dyadic processes and features related to COVID-19 fear during the transition to parenthood. While our findings highlight the significant role of couples’ perceived consensus and reflective functioning in managing these fears, we must acknowledge that our sample did not include partner-reported data. This omission restricts our ability to directly assess the dynamics of agreement or disagreement between partners, which are crucial elements in understanding the full spectrum of dyadic interactions during such a critical period. Future studies are recommended to involve both partners in the research process, thereby providing a more comprehensive analysis of how mutual perceptions within couples influence psychological outcomes during pregnancy. Furthermore, although the sample size meets the minimum requirements for detecting mediated effects, it may still be considered small for generalizing to a broader population. Moreover, the study’s design did not include comparison groups, focusing instead on internal dynamics within a specific cohort of primiparous future mothers. This approach limits our ability to draw causal inferences and compare these findings against a clinical group (e.g., depressed mothers), which might have provided additional validation of the observed effects. Future research could address these limitations by incorporating larger, more diverse samples and designing studies that include comparison or control groups to enhance the robustness and applicability of the findings.

## 5. Conclusions

Our study underscores the significant role of couples’ perceived consensus by mothers, reflective functioning, and the modulation of maternal perinatal affective difficulties in mitigating the fear of COVID-19 among pregnant women. This research highlights the urgent need to focus on relational dynamics in interventions designed to support maternal mental health during pregnancy. Specifically, enhancing mentalization abilities and promoting affective regulation could effectively reduce the psychological impacts associated with pandemic-related stressors. These insights pave the way for developing future interventions that aim to support expectant mothers by reducing feelings of fear and isolation while also addressing perinatal maternal affective challenges. In clinical practice, healthcare providers are encouraged to incorporate screening for couple dynamics, perinatal affectivity, and reflective functioning into routine prenatal care assessments. This approach should evaluate the quality of communication and consensus within the couple, alongside the mentalization abilities of expectant mothers. The development and implementation of intervention programs that focus on strengthening couple relationships and improving maternal reflective functioning are recommended. Furthermore, health policymakers should consider these findings when designing public health campaigns and policies aimed at pregnant populations, ensuring that these policies support not only the physical but also the mental health of expectant mothers by promoting stable and supportive relational environments. Additionally, further research should explore the longitudinal effects of couple dynamics on maternal mental health beyond the prenatal period to understand the long-term benefits of interventions aimed at enhancing couple consensus, dyadic coping, emotion regulation, and reflective functioning. Expanding the demographic scope to include diverse populations would enrich the understanding and applicability of these findings, thereby improving mental health outcomes for mothers and influencing the developmental trajectories of children born during these challenging times.

## Figures and Tables

**Table 1:** Descriptive statistics.

						Skewness		Kurtosis	
	Mean	Median	SD	Minimum	Maximum	Skewness	SE	Kurtosis	SE
RF	20.87	20.00	7.99	7	45	0.604	0.125	−0.0600	0.249
CONSENSUS	56.08	57.00	5.62	35	65	−0.678	0.125	0.2665	0.249
PMA	5.36	5.00	3.29	0	16	0.832	0.125	0.2730	0.249
FOC19	5.96	5.00	5.37	0	22	0.825	0.125	−0.1275	0.249

Note: RF = reflective functioning (Reflective Functioning Questionnaire; RFQ-8); CONSENSUS = dyadic consensus (Dyadic Adjustment Scale; DAS); PMA = perinatal maternal affectivity (Perinatal Assessment of Maternal Affectivity; PAMA); FOC19 = fear of COVID-19 (Multidimensional Assessment of COVID-19-Related Fears; MAC-RF).

**Table 2:** Correlation matrix.

	FOC19	PMA	CONSENSUS	RF
FOC19	-			
PMA	0.180 ***	-		
CONSENSUS	−0.118 *	−0.466 ***	-	
RF	0.185 ***	0.386 ***	−0.347 ***	-

Note. * *p* < 0.05, ** *p* < 0.01, *** *p* < 0.001. RF = reflective functioning (Reflective Functioning Questionnaire; RFQ-8); CONSENSUS = dyadic consensus (Dyadic Adjustment Scale; DAS); PMA = perinatal maternal affectivity (Perinatal Assessment of Maternal Affectivity; PAMA); FOC19 = fear of COVID-19 (Multidimensional Assessment of COVID-19-Related Fears; MAC-RF).

**Table 3:** Indirect and total effects of the mediation model.

				95% C.I.				
Type	Effect	Estimate	SE	Lower	Upper	β	z	*p*
Indirect	CONSENSUS → PMA → FOC19	−0.0537	0.0256	−0.1040	−0.0035	−0.0565	−2.095	0.036
	CONSENSUS → RF → FOC19	−0.0441	0.0187	−0.0807	−0.0076	−0.0464	−2.366	0.018
Component	CONSENSUS → PMA	−0.2731	0.0265	−0.3251	−0.2212	−0.4663	−10.301	<0.001
	PMA → FOC19	0.1967	0.0919	0.0165	0.3768	0.1211	2.139	0.032
	CONSENSUS → RF	−0.4931	0.0682	−0.6267	−0.3595	−0.3471	−7.233	<0.001
	RF → FOC19	0.0895	0.0358	0.0194	0.1596	0.1337	2.504	0.012
Direct	CONSENSUS → FOC19	−0.0152	0.0567	−0.1262	0.0959	−0.0159	−0.268	0.789
Total	CONSENSUS → FOC19	−0.1130	0.0486	−0.2082	−0.0179	−0.1184	−2.328	0.020

Note: RF = reflective functioning (Reflective Functioning Questionnaire; RFQ-8); CONSENSUS = dyadic consensus (Dyadic Adjustment Scale; DAS); PMA = perinatal maternal affectivity (Perinatal Assessment of Maternal Affectivity; PAMA); FOC19 = fear of COVID-19 (Multidimensional Assessment of COVID-19-Related Fears; MAC-RF).

## Data Availability

The data presented in this study are available on request from the corresponding author due to privacy’s and ethical reasons.
